# RWHMDA: Random Walk on Hypergraph for Microbe-Disease Association Prediction

**DOI:** 10.3389/fmicb.2019.01578

**Published:** 2019-07-10

**Authors:** Ya-Wei Niu, Cun-Quan Qu, Guang-Hui Wang, Gui-Ying Yan

**Affiliations:** ^1^School of Mathematics, Shandong University, Jinan, China; ^2^Data Science Institute, Shandong University, Jinan, China; ^3^Academy of Mathematics and Systems Science, Chinese Academy of Sciences, Beijing, China

**Keywords:** hypergraph, random walk, microbe, human diseases, association prediction

## Abstract

Based on advancements in deep sequencing technology and microbiology, increasing evidence indicates that microbes inhabiting humans modulate various host physiological phenomena, thus participating in various disease pathogeneses. Owing to increasing availability of biological data, further studies on the establishment of efficient computational models for predicting potential associations are required. In particular, computational approaches can also reduce the discovery cycle of novel microbe-disease associations and further facilitate disease treatment, drug design, and other scientific activities. This study aimed to develop a model based on the random walk on hypergraph for microbe-disease association prediction (RWHMDA). As a class of higher-order data representation, hypergraph could effectively recover information loss occurring in the normal graph methodology, thus exclusively illustrating multiple pair-wise associations. Integrating known microbe-disease associations in the Human Microbe-Disease Association Database (HMDAD) and the Gaussian interaction profile kernel similarity for microbes, random walk was then implemented for the constructed hypergraph. Consequently, RWHMDA performed optimally in predicting the underlying disease-associated microbes. More specifically, our model displayed AUC values of 0.8898 and 0.8524 in global and local leave-one-out cross-validation (LOOCV), respectively. Furthermore, three human diseases (asthma, Crohn’s disease, and type 2 diabetes) were studied to further illustrate prediction performance. Moreover, 8, 10, and 8 of the 10 highest ranked microbes were confirmed through recent experimental or clinical studies. In conclusion, RWHMDA is expected to display promising potential to predict disease-microbe associations for follow-up experimental studies and facilitate the prevention, diagnosis, treatment, and prognosis of complex human diseases.

## Introduction

Microbes exist in almost all habitats of flora and fauna, including humans. Deeper microbiological insights have indicated more compact associations between humans and their microflora (Sommer and Backhed, 2013). Some microbes are harmless and vital for host health in various manners, such as enhancement of host immunity, improvement of host metabolic capability, and protection of the host against pathogens (Eckburg et al., 2003; Ventura et al., 2009). Over the past few decades, numerous studies have focused on microbes inhabiting humans (Peterson et al., 2009). For instance, the gut flora are a complicated microbial community in the human digestive tract (Sommer and Backhed, 2013). Human gut microbes potentially benefit the host by synthesizing different vitamins, metabolizing bile acids, etc., thus exhibiting a fundamentally mutualistic association between some gut flora and the human host (Clarke et al., 2014). Therefore, microbes may be considered a supplemental “organ” in the host (Bäckhed et al., 2005). Furthermore, the number of microbial cells in the human body is reportedly approximately 10-fold the number of human cells (Rosner, 2014). Therefore, it is essential to systematically analyze associations between microbes and humans. The Human Microbiome Project (HMP) has furthered the current understanding of microbial structure, diversity, and function over the years (Human Microbiome Project Consortium, 2012). However, numerous basic and clinical studies have investigated the association between the human microbiome and human health (Moore and Moore, 1995; Dethlefsen et al., 2007; Zhang et al., 2009; Brown et al., 2011).

It is important to understand microbe-host interactions, which could benefit the prevention, diagnosis, treatment, and prognosis of human diseases (Bao et al., 2017; Zou et al., 2018). Microbial communities could be influenced by not only maternal genetic factors (Khachatryan et al., 2008; Turnbaugh et al., 2009; Goodrich et al., 2014) but also the habitat environments, such as the change of season (Davenport et al., 2014), host diet (David et al., 2014), antibiotic consumption (Donia et al., 2014), host smoking habits (Mason et al., 2015), and residential hygiene of the host (Sommer and Backhed, 2013). Changes in environmental variables may modify microbial communities and alter host-microbe interactions (Ma et al., 2014). In the past decades, with the development of high-throughput sequencing techniques and ensuing computational tools, increasing evidence demonstrates the close association between microbial dysbiosis and various human diseases (Neish, 2009), such as inflammatory bowel disease (IBD) (Frank et al., 2007), diabetes (Brown et al., 2011; Giongo et al., 2011), asthma (Chen and Blaser, 2007), obesity (Ley et al., 2006), and some cancers (Moore and Moore, 1995; Schwabe and Jobin, 2013). For example, through 16S rRNA microarray and parallel clone library-sequencing analysis, Huang et al. (2011) collected bronchial epithelial brushings from 65 asthma patients and compared them with 10 other samples from healthy control subjects, reporting that members of the airway microbiota, such as *Comamonadaceae*, *Sphingomonadaceae*, and *Oxalobacteraceae*, were greater in asthma patients. Hoppe et al. (2011) evaluated the effect of *Oxalobacter formigenes* on primary hyperoxaluria, a rare genetic disease. In particular, the urinary oxalate test and *ad hoc* analysis in their study revealed a reduction in *Oxalobacter formigenes* in patients with kidney stones. Furthermore, to analyze and elucidate the microbiota of colon cancer patients, Sobhani et al. (2011) extracted bacterial DNA from 179 colon cancer patients. Through qPCR and the immunohistochemical analyses, *C. coccoides*, *Bacteroides*, *Lactobacillus groups*, and *Faecalibacterium prausnitzii species* were reportedly increased in colon cancer patients. Moreover, on comparing microbes from 83 healthy control individuals and 98 liver cirrhosis patients, Qin et al. (2014) identified several biomarkers associated with liver cirrhosis, reporting that certain groups were reduced (e.g., *Alistipes finegoldii*, *Bacteroides eggerthii*, and *Coprococcus*) while certain others were enriched (e.g., *Fusobacterium*, *Haemophilus parainfluenzae*, and *Phascolarctobacterium*). Therefore, elucidation of the association between microbes and human diseases may facilitate novel drug discovery.

Despite some reported microbe-disease associations, they are not sufficient to completely understand disease pathogenesis, diagnosis, and treatment. Fortunately, Wang et al. (2015) proposed the excellent work about cancer hallmark network framework in the predictive genomics. The cancer hallmark network framework offered great insights on modeling genome sequencing data to predict cancer evolution and associated clinical phenotypes, which provided valuable designment strategies for using the framework in conjunction with genome sequencing data in any other attempt to prediction works on human diseases, drug targets and other fields, microbe included. Indeed, construction of a computationally efficient model from existing associations to predict potential ones is practical, potentially providing novel insights into time-consuming microbiology experiments by elucidating the most promising previously unknown associations (Chen et al., 2017c). Specifically, in determining lncRNA-disease associations (Chen, 2015), studies on drug targets (van Laarhoven et al., 2011; Yamanishi, 2013) and miRNA-disease associations (Wang et al., 2010; Chen et al., 2017d) have yielded various efficient *in silico* models to predict the underlying associations. Recently, based on experimentally verified microbe-disease associations, Ma et al. (2017) constructed the first Human Microbe-Disease Association Database (HMDAD). Thereafter, several computational models have been proposed to further contribute to the HMDAD. For example, Chen et al. (2017a) generated a model based on the KATZ measure, named KATZHMDA. In their model, they first constructed an association network showing pairwise relationships between microbes and human disease. Furthermore, they introduced Gaussian interaction profile kernel similarity for microbes and diseases to predict novel associations. Moreover, Huang Z.A. et al. (2017) developed a model of Path-Based Human Microbe-Disease Association Prediction (PBHMDA), wherein they used a special depth-first search algorithm on the heterogeneous biological network. In particular, they investigated all possible paths between diseases and microbes to infer highly probable associations. Resulting from the idea of collaborative recommendation model, Huang Y.A. et al. (2017) provided a computational model by adopting neighbor-based collaborative filtering and a graph-based scoring approach to calculate the association possibility of unknown microbe–disease pairs. The usage of hybrid approach based on two single recommendation methods contributed much more on their prediction results. Based on the microbe-disease interaction network, Wang et al. (2017) developed the model of Laplacian Regularized Least Squares for Human Microbe-Disease Association (LRLSHMDA). LRLSHMDA is a semi-supervised computational model using the Laplacian regularized least squares classifier. Recently, Zou et al. (2018) integrated symptom-based disease similarity to predict novel human microbe-disease associations based on network consistency projection (NCPHMDA). In detail, they conducted microbe space projection and disease space projection and combined the projections to design an advisable non-parametric approach. Based on adaptive boosting approach, Peng et al. (2018) developed a model named Adaptive Boosting for Human Microbe-Disease Association prediction (ABHMDA) to reveal the underlying associations between microbes and human diseases by calculating the association probability of concerned disease-microbe pair by grouped weak classifiers to form a stronger classifier for further scoring and sorting samples. Not long time ago, Qu et al. (2019) proposed a computational model on the basis of HMDAD by the methods of matrix decomposition and label propagation, which divided the original adjacency matrix about the relationship between microbes and diseases into a linear combination of itself and a low-rank matrix to predict novel disease-microbe associations.

Herein, we present a Random Walk on Hypergraph for Microbe-Disease Association Prediction (RWHMDA) model to predict underlying microbe-disease associations. In particular, we constructed a higher-order hypergraph model to accurately determine the implicit inherent association between microbes and human diseases. Thereafter, we generalized the well-known random walk process to the hypergraph in a modified manner, wherein vertices (microbes) within a hyperedge (human disease) were differentiated by the walker depending on their features. Finally, we ranked all candidate microbes for every investigated human disease. The merit of this study is the introduction of the concept and method of hypergraph to predict microbe-disease associations. Hypergraph is practical and suitable because it could provide biologically decipherable aspects by placing all disease-associated microbes in one hyperedge. Furthermore, we implemented global and local Leave-one-out cross-validation (LOOCV) to evaluate the predictive performance of RWHMDA.

## Materials and Methods

### Human Microbe-Disease Associations

In this study, we utilized microbe-disease associations in HMDAD database (Ma et al., 2017)^1^, containing 483 known microbe-disease associations among 292 microbes inhabiting the human body and 39 human diseases. The associations in HMDAD were obtained from sequencing-based microbiological analyses. In addition, if different data are available for overlapping microbe-disease associations in the database, only one record would be maintained. Finally, we obtained 450 distinct known microbe-disease associations for further prediction. Microbe-disease associations could be stored in an adjacency matrix*A*, where element *A*(*i, j*) represented the binary association of disease *d*(*i*) and microbe *m*(*j*). In other words, we obtained a *nd* × *nm* matrix *A*, where 450 elements were 1 and the others were 0. Meanwhile, *nd* was the number of diseases, and *nm* was the number of microbes.

### Gaussian Interaction Profile Kernel Similarity for Microbes

Gaussian interaction profile kernel similarity was calculated on the basis of a type of Radial Basis Function (RBF), namely Gaussian kernel function. In this study, we adopted the Gaussian interaction profile kernel similarity to determine the similarity between microbes. In detail, based on the constructed adjacency matrix *A*, microbial interaction profiles could be defined as a binary vector IP(*m*(*j*)), representing the absence or presence of the interaction between microbe *m*(*j*) and diseases. IP(*m*(*j*)) was the *j*-th column of matrix *A*. Thereafter, we calculated the Gaussian kernel similarity between microbe *m*(*j*) and microbe *m*(*j*), using Gaussian kernel function as follows:

(1) $$G\,M\,\left(m\,\left(i\right),m\,\left(j\right)\right)=\exp\left(-r_{m}\,{||I\,P\,\left(m\,\left(i\right)\right)-I\,P\,\left(m\,\left(j\right)\right)||}^{2}\right)$$

where *r*_*m*_ was set to balance the kernel bandwidth, and *GM* defined the Gaussian interaction profile kernel similarity matrix for microbes. Specially, *r*_*m*_was calculated in accordance with a new parameter *r*_*m*_ and the average known association number per miRNA as follows:

(2) $$r_{m}=\frac{r_{m}^{\prime }}{\left(\frac{1}{n\,m}\,\sum_{i=1}^{n\,m}{||I\,P\,\left(m\,\left(i\right)\right)||}^{2}\right)}$$

where *nm* is the total number of microbes. Technically, *r*_*m*_ was set as 1 here (Chen et al., 2017b).

### RWHMDA

**FIGURE 1:**
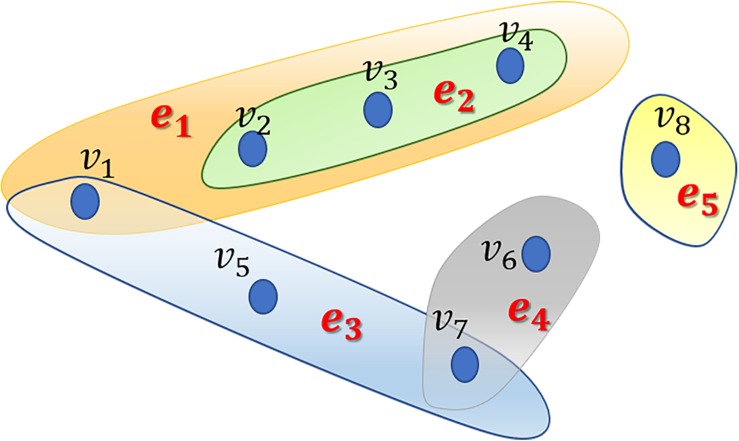
An example of a hypergraph comprising 5 hyperedges and 8 nodes. Different hyperedges are indicated with different colors. Every hyperedge contains different numbers of nodes based on their practical applications.

In this study, we proposed the RWHMDA model from the random walk on hypergraph to predict novel microbe-disease associations. Although Gaussian interaction profile kernel similarity for microbes is also accounted for in this method, RWHMDA is still a graph structure-based model without extra domain information in microbiological studies. Random walks on simple graphs have been investigated extensively in various biological fields. However, random walks on hypergraph have not been reported with respect to the prediction of microbe-disease associations thus far. Hypergraph is a type of higher-order graphical representation of biological data, compensating for information loss in the normal graph method, exclusively describing pair-wise association structures (Figure 1).

Generally, in the present model, we first constructed a hypergraph comprising microbes and diseases, wherein diseases are presented as hyperedges and microbes are presented as nodes. If several microbes have been confirmed to be associated with one disease, they would be presented as nodes in the hyperedge corresponding to the disease. In the hypergraph, hyperedges can join numerous vertices (not limited to two nodes as in simple graph). Specifically, if microbe *m*(*i*) is associated with disease *d*(*j*), then node *m*(*i*) belongs to hyperedge *d*(*j*). Obviously, one microbe might belong to different hyperedges. We assessed all known microbe-disease associations and established a hypergraph with 39 hyperedges. Without loss of generalizability, we defined the hypergraph as *HG*⁢(*V*,ℰ) where ℰ is the set of hyperedges, and _*V*_ is the set of vertices. Hyperedge *e* ∈ ℰ is a subset of *V*, hyperedge *e* is incident with node *v* if the node belongs to the hyperedge. Neighborhood relationships among nodes can be defined if *v*∈*e*, *w*∈*e*.

**FIGURE 2:**
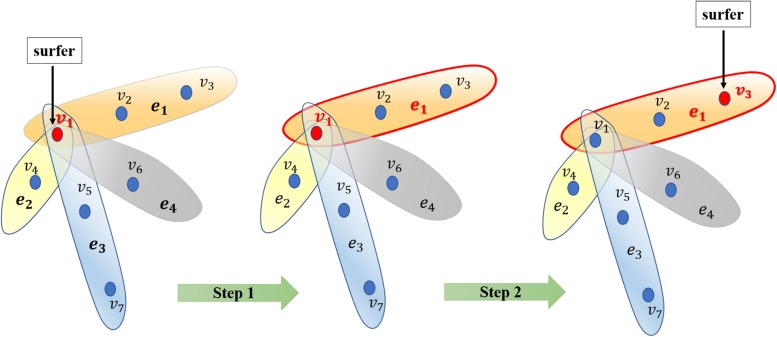
Illustration of the random walk process on the hypergraph. Generally, the surfer selects a hyperedge in step 1 and then selects a node as the destination vertex to shift to the selected hyperedge in step 2.

After constructing the hypergraph, we implement random walk with restart on it. The random walk on the normal graph is a type of Markov process. The surfer travels between nodes in the graph by starting at a node and shifting to an adjacent node at each discrete time step *t*. The transition probability between nodes is completely independent of the time *t*. Therefore, we could define the transition probability matrix *P* ∈ *R*^|*V*|×|*V*|^ for the whole process. Matrix *P* represents the transition probabilities of the random internodal movements. Matrix *P* is actually a critical factor calculated on the basis of multifarious filed knowledge. Furthermore, we introduced the random walk on the hypergraph. Basically, the surfer shifts between two nodes only if they are neighbors in the currently visited hyperedge. Briefly, this process may be considered a two-step procedure as follows: the surfer randomly selects a hyperedge incident with a currently visited node in step 1; thereafter, the surfer selects a destination neighbor node within the selected hyperedge in step 2 (Figure 2). Thereafter, we would focus on capturing transition matrix *P* with respect to the random walk on the hypergraph.

Considering an unweighted hypergraph *HG*⁢(*V*,ℰ), wherein hyperedges and nodes have no weights, the incidence matrix *H* ∈ *R*^|*V*|×|*E*|^ was defined as follows:

(3) $$h\,\left(v,e\right)=\left\{ \begin{array}{c} 1,i\,f\,v\in e\\ 0,i\,f\,v\notin e \end{array}\right.$$

(4) δ⁢(e)=|e|

(5) d⁢(v)=|ℰ⁢(v)|

where δ⁢(*e*) is the degree of hyperedge *e*, *d*(*v*) is the degree of vertex *v*, |*e*| indicates the number of nodes within hyperedge *e*, ℰ⁢(*v*) is the set of hyperedges incident with vertex *v*. Thereafter, we obtained the diagonal hyperedge degree matrix *D*_*e*_ ∈ *R*^|*E*|×|*E*|^, the diagonal vertex degree matrix *D*_*v*_ ∈ *R*^|*V*|×|*V*|^.

Regarding data on microbe-disease associations, it means the surfer would select a disease known to be associated with the current microbe. We could not unambiguously distinguish the more critical disease associated with the referenced microbe. Therefore, we intend for the surfer to uniformly randomly select a hyperedge at step 1. Furthermore, the surfer would walk to a node within this hyperedge. In our predictive case, although it is potentially difficult to evaluate the features of nodes, we differentiated microbes within a hyperedge of disease in accordance with the Gaussian interaction profile kernel similarity. Technically, in step 2, we intend for the surfer to shift to a node within a hyperedge in accordance with the sum of similarities of the node with all other nodes in the hypergraph. In summary, starting from node *u*, the surfer would select hyperedge *e* incident with *u* proportional to the weight of hyperedge *w*(*e*). Thereafter, the surfer selects node *v* proportional to the weight of *v* within the current hyperedge *e*, namely *w*⁢(*v*_*e*_).

Considering the afore-mentioned motivation, we then defined the weighted incident matrix *W* ∈ *R*^|*V*|×|*E*|^of hypergraph *HG*⁢(*V*,ℰ) as follows:

(6) $$w\,\left(v,e\right)=\left\{ \begin{array}{cc} w\,\left(v_{e}\right), & i\,f\,v\in e\\ 0, & i\,f\,v\notin e \end{array}\right.$$

where *w*⁢(*v*_*e*_) is the weight of node *v* in hyperedge *e*. In the present model, we calculated *w*⁢(*v*_*e*_) on the basis of matrix *GM.* In this study, the weight of a microbe *m*(*i*) in a hyperedge is the sum of *i*th row in *GM*. Thereafter, we redefined hyperedge degree δ′⁢(*e*) and hyperedge degree matrix *D*_*ve*_ ∈ *R*^|*E*|×|*E*|^ as follows:

(7) $$\delta \,\left(e\right)=\sum_{v\in e}w\,\left(v,e\right)$$

where *D*_*ve*_ is the diagonal hyperedge degree matrix with element δ⁢(*e*).

We then calculated the transition probability from vertex *u* to vertex *v* as follows:

(8) $$P\,\left(u,v\right)=\sum_{e\in E}w\,\left(e\right)\,\frac{h\,\left(u,e\right)}{\sum_{\hat{e}\in ℰ}w\,\left(\hat{e}\right)}\,\frac{w\,\left(v,e\right)}{\sum_{\hat{v}\in e}w\,\left(\hat{v},e\right)}$$

which may also be expressed in matrix form as follows:

(9) $$P=D_{v}^{-1}\,H\,W_{e}\,D_{v\,e}^{-1}\,W^{T}$$

where *W*_*e*_ ∈ *R*^|*E*|×|*E*|^ is the diagonal matrix of hyperedge weights, wherein all diseases are considered with equal weightage in accordance with the previously described practical consideration, i.e., all hyperedges are 1/|*E*|. Naturally, transition matrix *P* is stochastic, implying that the sum of every row equals 1.

Furthermore, we implement the random walk with restart on the hypergraph. In particular, assuming that microbes associated with disease *d*(*i*) are to be predicted, all microbes with known associations with *d*(*i*) are considered seed microbes, while the others are considered candidate microbes. Thereafter, we set the initially normalized probability vector $\vec{v}\,\left(0\right)\in R^{\left|V\right|\times 1}$ such that seed microbes are assigned with equal probability and the non-seed miRNAs are zero. After the first step, $\overset{\to }{\underset{¯}{v}}\,\left(1\right)=P^{T}\,\vec{v}\,\left(0\right)$. Moreover, we set the restart probability at every step at source nodes as α(0 < α < 1), $\vec{v}\,\left(1\right)=\left(1-\alpha \right)\,P^{T}\,\vec{v}\,\left(0\right)+\alpha \,\vec{v}\,\left(0\right)$. Finally, we obtained the random walk with the following formula:

(10) $$\vec{v}\,\left(t+1\right)=\left(1-\alpha \right)\,P^{T}\,\vec{v}\,\left(t\right)+\alpha \,\vec{v}\,\left(0\right)$$

**FIGURE 3:**
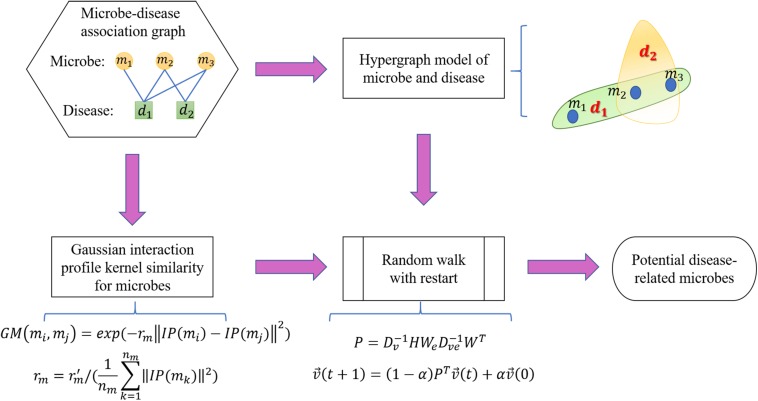
Schematic representation of the RWHMDA model.

$\vec{v}\,\left(t\right)$ is defined such that the *i*th element means the probability of moving to node *i* at step *t*. After some steps, the random walk would stabilize, implying that the difference between $\vec{v}\,\left(t+1\right)$ and $\vec{v}\,\left(t\right)$ measured by the L1 norm is smaller than the provided threshold. The stable state of the random walk with restart is defined as $\vec{v}\,\left(\infty \right)$. Stationary probability in $\vec{v}\,\left(\infty \right)$ indicates the probable associations between candidate microbes with the currently investigated disease. We conducted the random walk for every disease in the HMDAD database and ranked the underlying microbe-disease associations in accordance with the corresponding $\vec{v}\,\left(\infty \right)$ of the current disease (Figure 3).

As a supplement, we set the α-value as 0.2 and set the cutoff value as 10^–6^.

## Results

### Performance Evaluation

**FIGURE 4:**
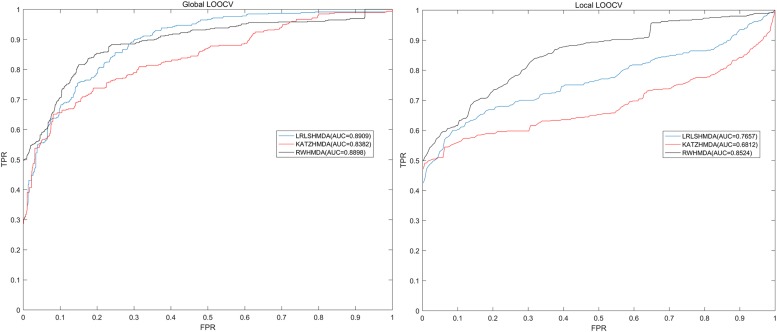
Comparisons between the RWHMDA model and the other two state-of-the-art prediction models (LRLSHMDA and KATAHMDA) in terms of global and local AUC values. Consequently, RWHMDA yielded AUCs of 0.8898 and 0.8524, yielding a better prediction performance.

LOOCV was usually implemented to assess the performance of the prediction model. Global and local LOOCV in the present study were both conducted to comprehensively assess the performance of RWHMDA. Specifically, global LOOCV was conducted on the basis of the known microbe-disease associations in the HMDAD database (Ma et al., 2017). Each association was left out in turn as the test sample, while others were set as candidate samples. If the rank of the test sample was higher than that of the candidate samples, the test association was considered to have been correctly predicted. Furthermore, local LOOCV was somewhat different from global LOOCV, and it was implemented as follows: first, for an investigated disease, based on the association records in the HMDAD (Ma et al., 2017) database, each known disease-associated microbe was excluded in turn as the test sample and the others were used as seed samples. Thereafter, the predicted association probability of the current test sample would be ranked with the probability of candidate samples. If the test sample was ranked beyond the threshold, the model successfully predicted this microbe–disease association. Further, we plotted a receiver operating characteristics (ROC) curve. The area under the ROC curve (AUC) was determined to assess the prediction performance of RWHMDA. Specifically, AUC = 1 implied an excellent performance, and AUC = 0.5 indicated a random performance. Consequently, RWHMDA yielded a global AUC value of 0.8898 and local AUC value of 0.8524, which were higher than some previously reported computational models, such as LRLSHMDA (0.8959, 0.7657) (Bao et al., 2017) and KATZHMDA (0.8382, 0.6812) (Chen et al., 2017a; Figure 4).

### Case Studies

To further assess the performance of the proposed model, we conducted case studies of asthma, Crohn’s disease (CD), and type 2 diabetes by assessing the 10 highest probable microbes ranked by RWHMDA.

It is unambiguous that the human microflora play an important role in asthma pathogenesis (Li N. et al., 2017). Morbidity rates among asthma patients have significantly increased since the 1960s (Anandan et al., 2010). Asthma caused approximately 400 thousand deaths worldwide in 2015. More recently, on evaluating data regarding the association between *Helicobacter pylori* status with the history of asthma from 7663 adults in the Third National Health and Nutrition Examination Survey, childhood acquisition of *H. pylori* is associated with a reduced risk of asthma (Chen and Blaser, 2007). We implemented RWHMDA for the asthma case study. Consequently, 9 of the 10 most highly ranked asthma-related microbes were confirmed from the literature (Table 1). For example, the study reporting the presence of *Propionibacterium acnes* (1st ranked in the prediction list of RWHMDA) in asthma patients helped diagnose asthma (Romero-Espinoza et al., 2018). *Pseudomonas*, ranked 3rd by our model, was confirmed to be more prevalent in the sputum of asthma patients (Jung et al., 2016). Moreover, as the 10th predicted asthma-related microbe, *Streptococcus* are associated with asthma, potentially contributing to its pathophysiology (Zhang et al., 2016).

**TABLE 1:** A case study on predicted potential asthma-related microbes.

**Rank**	**Microbe**	**Evidence**
1	*Propionibacterium acnes*	PMID:29447223
2	*Propionibacterium*	PMID:29447223
3	*Pseudomonas*	PMID:27433177
4	*Burkholderia*	PMID:24451910
5	*Enterobacter aerogenes*	PMID:18790035
6	*Enterobacter hormaechei*	Unconfirmed
7	*Klebsiella pneumoniae*	PMID:26220531
8	*Shigella dysenteriae*	Unconfirmed
9	*Actinobacteria*	PMID:23265859
10	*Streptococcus*	PMID:27078029

**Among the 10 highest ranked potential asthma-related microbes, eight were confirmed from the literature.**

The worldwide prevalence of diabetes mellitus has increased continuously over the past few decades (Tadic and Cuspidi, 2015). Type 2 diabetes mellitus is a subclass of diabetes mellitus, accounting for approximately 90% of all the diabetes mellitus cases. The traditional view holds that the pathogenesis of type 2 diabetes is associated with both genetic and lifestyle-related factors. Recent evidence suggests that the pathomechanism and pathogenesis of type 2 diabetes mellitus are also associated with an unbalance in microbial communities (Ripsin et al., 2009; Furet et al., 2010). Larsen et al. (2010) assessed the differences in the composition of the intestinal microbiota in individuals without and those with type 2 diabetes via high-throughput 16S rDNA gene pyrosequencing, reporting an increase in *Bacilli*, *Bacteroidetes*, and *Betaproteobacteria* and reductions in *Clostridia*, *Clostridium*, *Firmicutes*, etc. Among the 10 highest ranked microbes by probability, 8 were confirmed through recent evidence (Table 2). For example, *Fusobacterium nucleatum* was ranked first and confirmed to be significantly higher in type 2 diabetes mellitus patients than in those without type 2 diabetes mellitus (Miranda et al., 2017). *Pseudomonas*, abundant in the subgingival plaque, ranked 2nd by our model and was markedly different between individuals with and those without diabetes (Zhou et al., 2013). Furthermore, *Aerococcus* and *Atopobium* were associated with the risk of type 2 diabetes (Li H. et al., 2017; Long et al., 2017).

**TABLE 2:** RWHMDA used to predict candidate microbes associated with type 2 diabetes.

**Rank**	**Microbe**	**Evidence**
1	*Fusobacterium nucleatum*	PMID:28198980
2	*Pseudomonas*	PMID:23613868
3	*Aerococcus*	PMID:28786059
4	*Atopobium*	PMID:28177125
5	*Atopobium vaginae*	unconfirmed
6	*Candidate division TM7*	unconfirmed
7	*Eggerthella*	PMID:26046242
8	*Gardnerella*	PMID:28316574
9	*Gardnerella vaginalis*	PMID:2131794
10	*Lactobacillus crispatus*	PMID:28608654

**Consequently, 8 of the 10 most probable microbes were experimentally confirmed through the relevant literature.**

Crohn’s disease (CD) is a type of IBD. Although the etiology of CD is generally believed to associated with the combination of immune, environmental, and bacterial factors, however, the precise etiology of CD is still unclear (Dessein et al., 2008; Stefanelli et al., 2008; Cho and Brant, 2011). In fact, no surgical treatment or pharmacotherapeutic methods have been reported to cure Crohn’s disease (Baumgart and Sandborn, 2012). Studies have increasingly investigated the bacterial factors associated with the etiology of CD. Gevers et al. (2014) reported that the increased abundance of *Fusobacteriaceae*, *Enterobacteriaceae*, *Pasteurellacaea*, and *Veillonellaceae* and the decreased abundance of *Clostridiales Erysipelotrichales*, and *Bacteroidales* are closely correlated with Crohn’s disease. A case study on Crohn’s disease revealed that the 10 most probable microbes were confirmed through recent researches (Table 3). For example, the two most promising microbes predicted by our model were *Clostridium difficile* and *Bacteroides fragilis*, both confirmed to be present at high levels in CD patients compared than in healthy individuals (Cojocariu et al., 2014; Zhou et al., 2016). Moreover, studies evaluating the association between disease status and gut microbiota in CD patients revealed that *Clostridium coccoides* (3rd place in the ranking list) was abundant in febrile patients presenting with remission in comparison with patients with active CD (Prosberg et al., 2016).

**TABLE 3:** A case study on Crohn’s disease verifying all 10 of the 10 most probable candidates of Crohn’s disease-related microbes.

**Rank**	**Microbe**	**Evidence**
1	*Clostridium difficile*	PMID:25599768
2	*Bacteroides fragilis*	PMID:27684872
3	*Clostridium coccoides*	PMID:27687331
4	*Bacilli*	PMID:29559804
5	*Betaproteobacteria*	PMID:27833911
6	*Lachnospiraceae*	PMID:26628508
7	*Clostridium*	PMID:29722832
8	*Prevotella*	PMID:28852861
9	*Alistipes finegoldii*	PMID:28877044
10	*Alistipes putredinis*	PMID:29311644

## Discussion and Conclusion

Accumulating falsifiable evidence indicates that microbial involvement is associated with disease pathogenesis in some cases. In this study, with data from microbiological studies, hypergraph theory, and other research areas, we introduced an *in silico* model named RWHMDA to predict underlying microbe-disease associations. Many previous computational models performed pairwise comparisons and illustrated microbe-disease associations as a normal graph. RWHMDA has been developed primarily on the basis of a hypergraph, thus compensating for the information loss issue by normal graph. Known microbe-disease associations in the HMDAD database and the Gaussian interaction profile kernel similarity for microbes were utilized to design a weighted hypergraph comprising microbes and diseases. Random walk with restart was implemented on the hypergraph for every disease to identify the potential disease-associated microbes. Both cross-validation and case studies on asthma, type 2 diabetes, and Crohn’s disease revealed the reliability of RWHMDA. In addition, the predicted microbes for all diseases were publicly released for further validation through biological assays (Supplementary Table S1).

Generally, RWHMDA performed reliably, thus revealing several important factors. First, as a representation of a higher-order structure, hypergraphs adequately illustrate and present data on microbe-disease associations without information loss. In particular, the practice of setting disease as a hyperedge and microbe as a node was reasonable and biologically decipherable, thereby naturally benefiting the prediction of potential associations. Second, owing to the valid and updated data on disease-microbe associations through numerous biological analyses, RWHMDA had a greater prediction accuracy with greater probability. Third, random walk process is a widespread and significant physical dynamic process, used extensively in numerous studies. RWHMDA was developed on the basis of the random walk with restart process, following a seemingly iterative 2-step walking strategy to investigate the potential association probability between any pair of microbe and disease.

However, the present RWHMDA model has some limitations. Hypergraph and Gaussian interaction profile kernel were both constructed largely on the basis of known associations. Therefore, the model may have a bias toward those well-known diseases and microbes. Furthermore, some other similarity measures of diseases could also be meticulously integrated into the RWHMDA model, such as symptom-based disease similarity and disease semantic similarity. Finally, the RWHMDA model could not be implemented for new diseases without known associations with microbes, being an inherent limitation of the graph-based model.

In conclusion, RWHMDA is expected to display promising potential to predict disease-microbe associations for follow-up experimental studies and facilitate the prevention, diagnosis, treatment, and prognosis of complex human diseases.

## Author Contributions

Y-WN developed the prediction method, conducted the experiments, analyzed the result, and wrote the manuscript. C-QQ, G-YY, and G-HW conceived the project, analyzed the result, and wrote the manuscript. All authors read and approved the final manuscript.

## Conflict of Interest Statement

The authors declare that the research was conducted in the absence of any commercial or financial relationships that could be construed as a potential conflict of interest.

## Footnotes

1 http://www.cuilab.cn/hmdad

## Supplementary Material

TABLE S1 We prioritized candidate microbes for all the investigated human diseases in the HMDAD database. The prediction results for each disease were publicly released for further validation. The relatively high ranked disease-microbe associations were anticipated to be confirmed by biological experiments or future clinical observation.
