# Supplementary material for: RWHMDA: Random Walk on Hypergraph for Microbe-Disease Association Prediction
Source: Front Microbiol. 2019 Jul 10;10:1578. doi: 10.3389/fmicb.2019.01578 (PMC6635699; doi:10.3389/fmicb.2019.01578)
Supplement: TABLE S1 — We prioritized candidate microbes for all the investigated human diseases in the HMDAD database. The prediction results for each disease were publicly released for further validation. The relatively high ranked disease-microbe associations were anticipated to be confirmed by biological experiments or future clinical observation. [file Table_1.docx]

Supplementary Material

**RWHMDA: Random Walk on Hypergraph for Microbe-Disease Association Prediction**

**Ya-Wei Niu^1^, Cun-Quan Qu^1,2^, Guang-Hui Wang^1,2,*^ and Gui-Ying Yan^3^**

^1^School of Mathematics, Shandong University, Jinan 250100, China

^2^Data Science Institute, Shandong University, Jinan 250100, China

^3^Academy of Mathematics and Systems Science, Chinese Academy of Sciences, Beijing 100190, China

***** **Correspondence:**

**Dr. Guang-Hui Wang**

Email: [ghwang@sdu.edu.cn](mailto:ghwang@sdu.edu.cn)

**Supplementary table.** We prioritized candidate microbes for all the human complex diseases recorded in HMDAD database. The predicted microbe ranks for each disease were publicly released for further experimental validation. The relatively high ranked disease-microbe associations were anticipated to be confirmed by biological experiments or future clinical observation.
